# Myxoid dermatofibrosarcoma protuberans of the medial thigh with perivulvar extension: A case highlighting diagnostic challenges and reconstructive planning

**DOI:** 10.1016/j.jdcr.2026.08.029

**Published:** 2026-08-25

**Authors:** Olivia N. Lamberg, Carmine Palladino, Spencer P. McClure, Brian L. Swick, Emma Killoran, Nicole A. Negbenebor

**Affiliations:** Department of Dermatology, University of Iowa, Iowa City, Iowa

**Keywords:** COL1A1::PDGFB fusion, dermatofibrosarcoma protuberans, Mohs micrographic surgery, molecular diagnosis, myxoid DFSP, perivulvar tumor, soft tissue sarcoma

## Introduction

Dermatofibrosarcoma protuberans (DFSP) is a rare, low-grade soft tissue sarcoma characterized by slow growth, local aggressiveness, and a high risk of recurrence if incompletely excised. It is most commonly driven by a COL1A1–platelet-derived growth factor subunit B (PDGFB) fusion, resulting in constitutive activation of platelet-derived growth factor signaling.^1^ Clinically, DFSP often presents as a long-standing, indurated plaque or nodular lesion with progressive enlargement.

Several histologic variants of DFSP have been described, including myxoid, pigmented (Bednar tumor), and fibrosarcomatous subtypes.^2^ Myxoid DFSP is an uncommon variant characterized by prominent myxoid stromal change (>50% myxoid stroma), which may obscure the classic storiform architecture and lead to diagnostic difficulty.^3^ As a result, it may mimic a range of benign and low-grade myxoid neoplasms, particularly on limited biopsy specimens.

Additionally, DFSP arising in the vulvar and perivulvar region poses unique surgical challenges due to the potential for broad subclinical extension and involvement of deeper high-risk functional structures.^4^ These anatomically sensitive locations require careful preoperative planning, detailed knowledge of regional anatomy, and multidisciplinary coordination for reconstruction.

Given these challenges, accurate diagnosis often requires integration of histopathologic findings with immunohistochemistry and molecular testing.^1^^,^^3^ We present a case of myxoid DFSP of the medial thigh with perivulvar extension, highlighting its diagnostic pitfalls, the importance of molecular confirmation, and the surgical considerations unique to this anatomically complex location.

## Case report

A 63-year-old woman presented following emergency department evaluation for an enlarging, intermittently bleeding mass on the left medial thigh and perivulvar area. The lesion had been present for more than 10 years, with gradual enlargement and episodic bleeding that progressed to ulceration 1 month prior to presentation. She denied fevers, chills, night sweats, unintentional weight loss, or lymphadenopathy.

Physical examination revealed a multinodular violaceous exophytic tumor with surface ulceration and adjacent subcutaneous nodularity on the left medial thigh extending to the perivulva. No palpable inguinal lymphadenopathy was appreciated (Fig 1).

**Fig 1:**
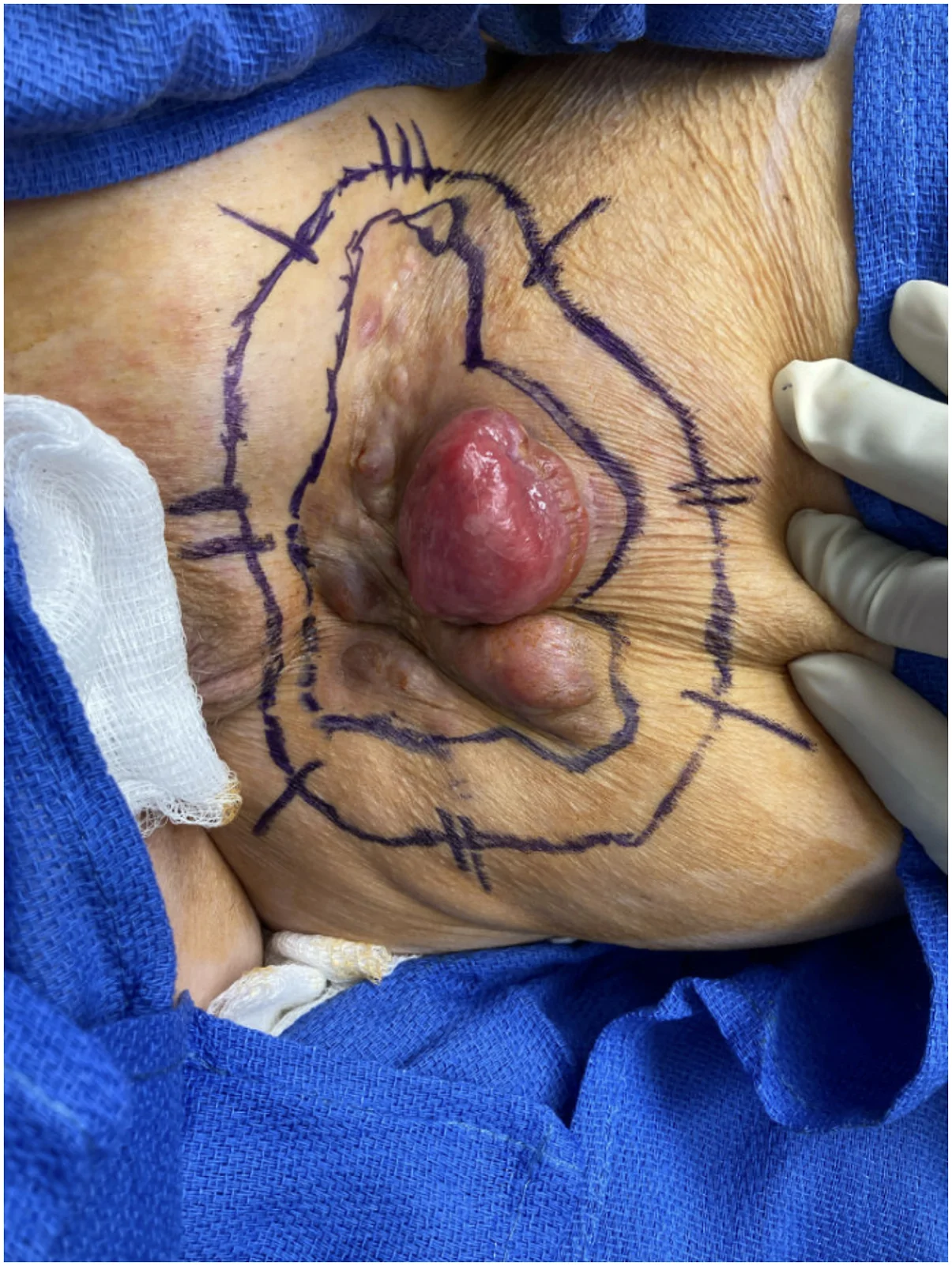
Preoperative clinical photograph demonstrating a multilobulated violaceous exophytic tumor of the left medial thigh with extension toward the perivulvar region and areas of surface ulceration.

Punch biopsy demonstrated a CD34-positive myxoid spindle cell neoplasm. Given the atypical histopathologic features, targeted RNA sequencing was performed, which identified a COL1A1::PDGFB fusion, confirming the diagnosis of dermatofibrosarcoma protuberans. Magnetic resonance imaging demonstrated a heterogeneous enhancing lesion extending from the medial thigh toward the perivulvar region, with both exophytic and infiltrative subcutaneous components and no definite muscular invasion. Prominent inguinal and pelvic lymph nodes were interpreted as reactive. Chest imaging showed no evidence of metastatic disease.

Given the tumor's location spanning the medial thigh, groin crease, and perivulvar region, multidisciplinary consultation with plastic surgery was obtained preoperatively. The anatomic considerations included proximity to the femoral neurovascular bundle, pudendal structures, and the need for durable soft tissue coverage across a high-motion intertriginous area.

The patient underwent Mohs micrographic surgery for definitive treatment. The preoperative tumor measured 8 × 3 cm and was debulked, with tissue submitted for permanent sections to evaluate for fibrosarcomatous transformation. Histopathologic evaluation demonstrated a spindle cell neoplasm with infiltrative growth through the dermis into the subcutis, with entrapment of adipocytes and prominent myxoid stroma (Fig 2). Tumor clearance was achieved after 2 stages. The final surgical defect measured 13 × 15 cm (Fig 3). Reconstruction was performed in coordination with plastic surgery. A medial thigh advancement flap was selected to provide durable, well-vascularized coverage across the groin crease and perivulvar region while minimizing donor site morbidity (Fig 4). This approach allowed for primary closure of the donor site and avoided the need for skin grafting in this high-motion area. The postoperative course was uncomplicated.

**Fig 2:**
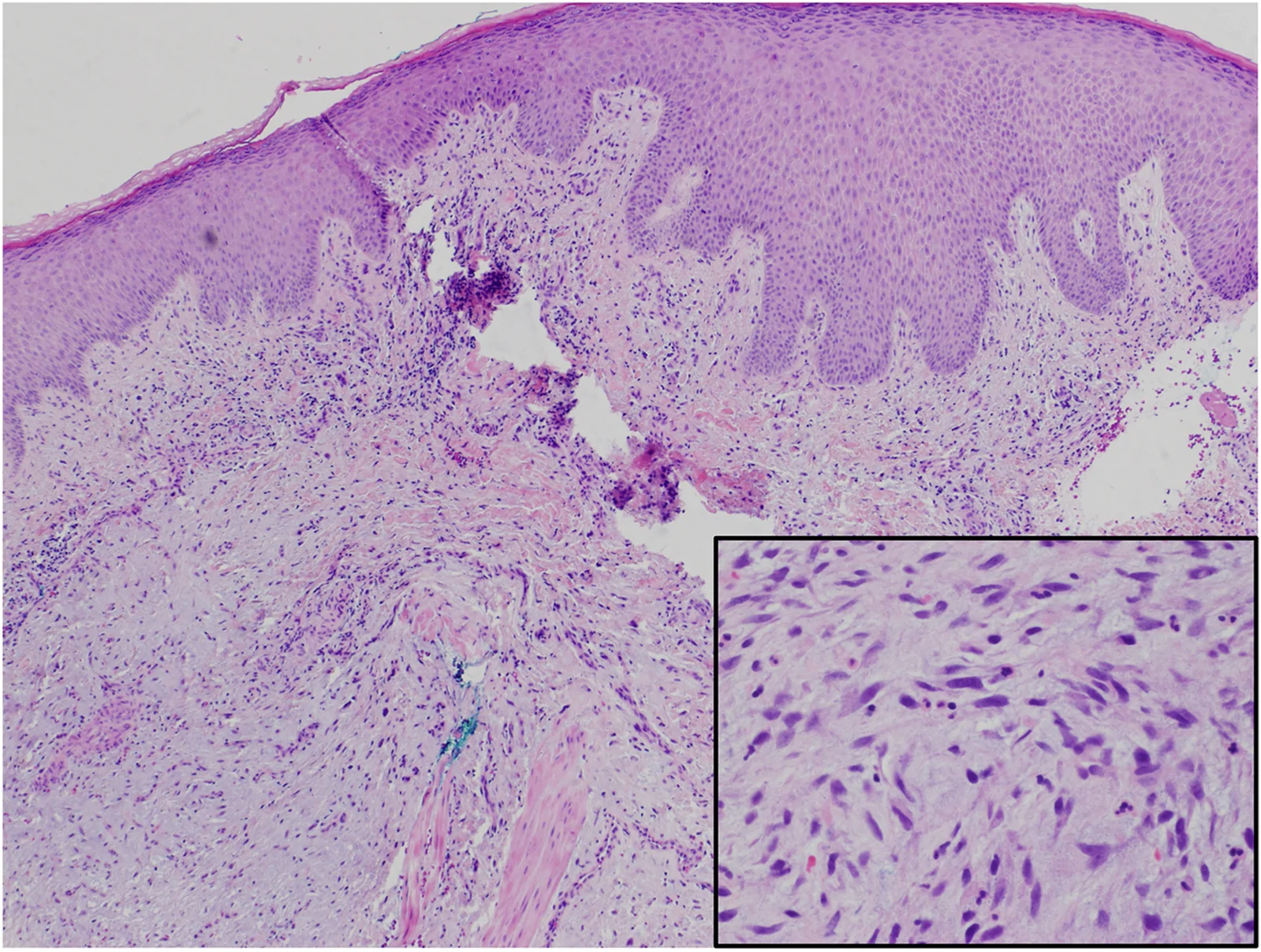
Skin biopsy histopathology demonstrating a spindle cell neoplasm within a prominent myxoid stroma with infiltrative growth into the subcutis and entrapment of adipocytes hematoxylin-eosin stain; 40× magnification with 400× inset).

**Fig 3:**
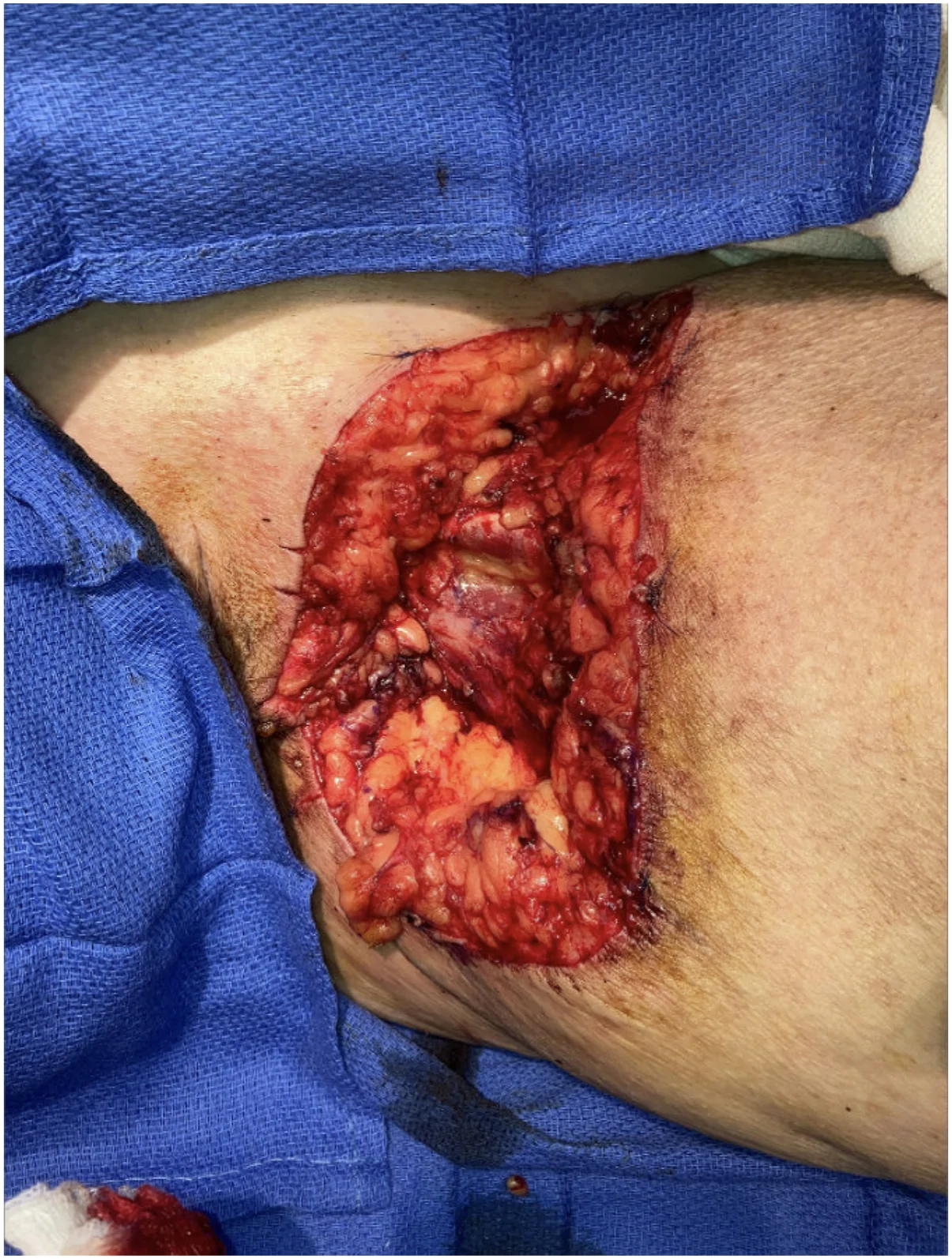
Postoperative defect following Mohs micrographic surgery demonstrating a large medial thigh and perivulvar wound prior to reconstruction.

**Fig 4:**
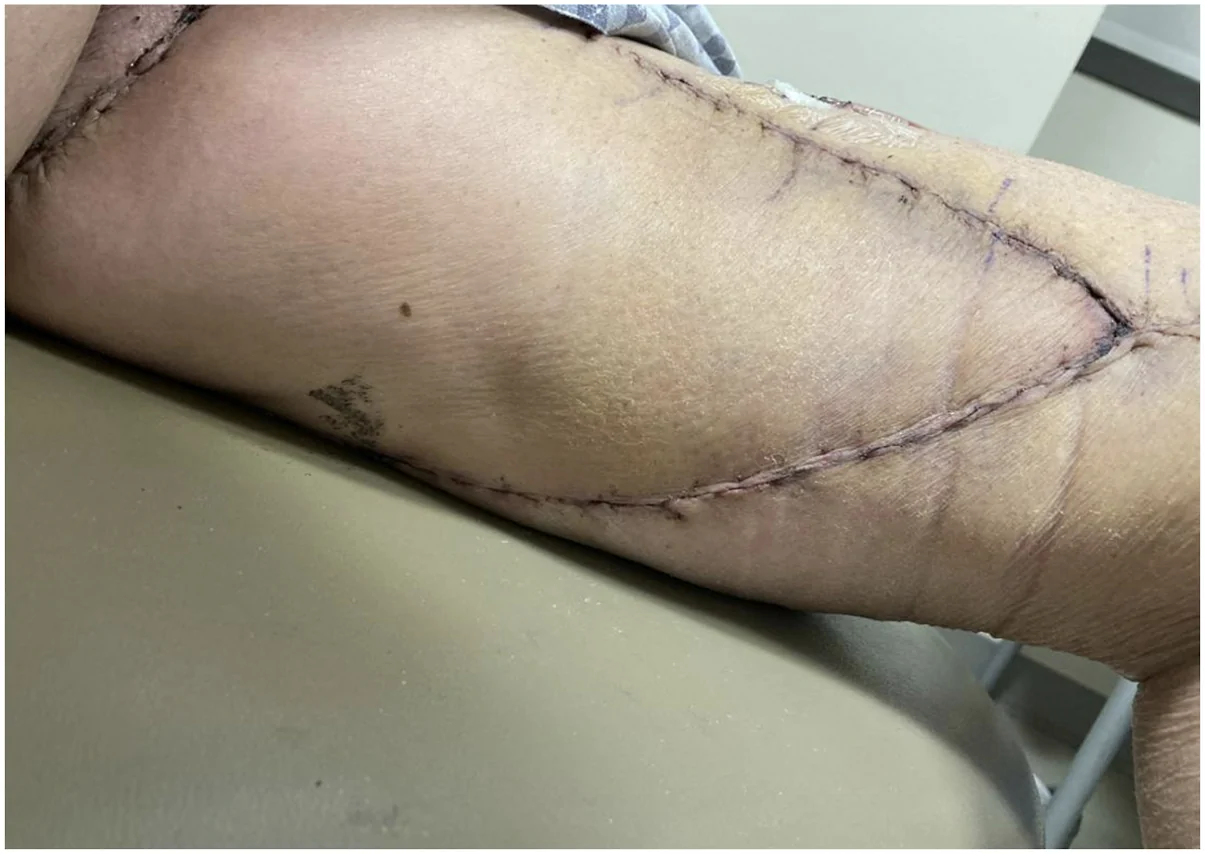
Postoperative reconstruction with a medial thigh advancement flap demonstrating durable soft tissue coverage of the medial thigh and perivulvar region.

## Discussion

Myxoid DFSP is an uncommon variant in which abundant myxoid stromal change may obscure the classic storiform growth pattern and infiltration of the tumor.^1^^,^^3^ This histologic variability can complicate diagnosis, particularly in small or superficial biopsy specimens, and may lead to misclassification as benign or low-grade myxoid neoplasms.^3^^,^^5^

In our case, the initial biopsy demonstrating a CD34-positive myxoid spindle cell neoplasm illustrates this diagnostic ambiguity. While CD34 expression supports the diagnosis of DFSP, it is not specific. The National Comprehensive Cancer Network (NCCN) guidelines recommend molecular testing for equivocal lesions.^6^ In our case, targeted RNA sequencing confirmed the diagnosis by demonstrating the hallmark COL1A1::PDGFB fusion and enabled appropriate surgical planning.^7^ Prior reports have similarly emphasized these pitfalls; a case of myxoid DFSP involving the vulva demonstrated initial misdiagnosis as a benign spindle cell neoplasm, with definitive diagnosis established only after detection of the COL1A1::PDGFB fusion.^8^

From a clinical standpoint, accurate diagnosis has important clinical implications, as DFSP is characterized by infiltrative growth and significant subclinical extension. In our case, the large final defect relative to the clinically apparent tumor (8 × 3 cm preoperatively versus 13 × 15 cm following excision) demonstrates significant subclinical spread. Margin-controlled techniques such as Mohs micrographic surgery allow for comprehensive peripheral and deep marginevaluation and have been associated with lower recurrence rates compared to wide local excision.^9^^,^^10^ A systematic review and meta-analysis found local recurrence rates of 1.5% after Mohs micrographic surgery compared to 9.4% after wide local excision in noncomparative studies.^10^ These advantages are particularly important in anatomically sensitive or functionally constrained regions such as the perivulva.

DFSP arising in the vulvar and perivulvar region poses unique surgical challenges and requires careful consideration of multiple factors, including defect size, exposure of critical structures, functional requirements, and the high-motion nature of the intertriginous location. Reconstructive options for groin and perivulvar defects include pedicled perforator flaps, regional muscle flaps, and local advancement techniques. In our case, a medial thigh advancement flap provided durable coverage across the groin crease while minimizing donor site morbidity.

Overall, this case highlights myxoid DFSP as an important diagnostic pitfall and reinforces the need for molecular confirmation in atypical presentations. Additionally, DFSP in anatomically complex locations requires careful preoperative planning and multidisciplinary coordination to achieve complete tumor clearance while optimizing functional outcomes.

## Conflicts of interest

None disclosed.

## References

[1] Llombart B., Serra-Guillén C., Monteagudo C., López Guerrero J.A., Sanmartín O. (2013). Dermatofibrosarcoma protuberans: a comprehensive review and update on diagnosis and management. Semin Diagn Pathol.

[2] Lim S.X., Ramaiya A., Levell N.J., Venables Z.C. (2023). Review of dermatofibrosarcoma protuberans. Clin Exp Dermatol.

[3] Mentzel T., Schärer L., Kazakov D.V., Michal M. (2007). Myxoid dermatofibrosarcoma protuberans: clinicopathologic, immunohistochemical, and molecular analysis of eight cases. Am J Dermatopathol.

[4] Reinhart J.P., Lachman N., Salinas-Alvarez Y., Demer A.M., Vidal N.Y. (2026). Vulvar dermatofibrosarcoma protuberans: surgical planning and management considerations for the dermatologic surgeon. Dermatol Surg.

[5] Reimann J.D., Fletcher C.D. (2007). Myxoid dermatofibrosarcoma protuberans: a rare variant analyzed in a series of 23 cases. Am J Surg Pathol.

[6] Bordeaux J, Blitzblau R, Aasi SZ (2025). Dermatofibrosarcoma protuberans, version 1.2025, NCCN Clinical Practice Guidelines in Oncology. J Natl Compr Canc Netw.

[7] Li N., Yang L., Zhang W. (2018). COL1A1-PDGFB gene fusion in dermatofibrosarcoma protuberans: a useful diagnostic tool and clinicopathological analysis. Oncol Lett.

[8] Wiszniewska J., Roy A., Masand R.P. (2016). Myxoid dermatofibrosarcoma protuberans of the vulva: case report of a rare variant in an unusual location, with unusual morphologic and immunohistochemical features. Am J Dermatopathol.

[9] Farma J.M., Ammori J.B., Zager J.S. (2010). Dermatofibrosarcoma protuberans: how wide should we resect?. Ann Surg Oncol.

[10] Snow S.N., Gordon E.M., Larson P.O., Bagheri M.M., Bentz M.L., Sable D.B. (2004). Dermatofibrosarcoma protuberans: a report on 29 patients treated by Mohs micrographic surgery with long-term follow-up and review of the literature. Cancer.

